# A Case Report on Initial Experience of Lattice Radiation Therapy for Managing Massive Non-small Cell Lung Cancer and Renal Pelvic Cancer

**DOI:** 10.7759/cureus.44764

**Published:** 2023-09-06

**Authors:** A Aziz Sait, Umang Patel, Jason Berilgen, Sunil Mani

**Affiliations:** 1 Department of Radiation Oncology, Millennium Physicians, The Woodlands, USA; 2 Faculty of Engineering, Teerthanker Mahaveer University, Moradabad, IND

**Keywords:** 3d grid radiation, renal cancer, nsclc, sfrt, lattice radiation therapy

## Abstract

Lattice radiation therapy (LRT) is an advanced treatment approach specifically designed for massive tumors. It aims to deliver high-dose regions within tumors while ensuring the safety of the surrounding dose-limiting organs at risk (OAR). This case report introduces two unique clinical cases: a 63-year-old male diagnosed with a massive non-small cell lung cancer (NSCLC) tumor and a 61-year-old male with an inoperable recurrent left-sided adrenal mass intricately surrounded by dose-limiting bowel structures. Both patients underwent LRT to enhance tumor control and maintain less toxicity. Notably, both patients displayed a significant tumor volume reduction accompanied by minimal adverse effects during the 12-month follow-up period. While these initial results suggest that LRT may be effective and safe for treating large tumors, further investigation through exhaustive research and multicenter trials is necessary to fully understand and determine the specifics of lattice radiation therapy techniques.

## Introduction

Radiation oncology has a long history of innovation and adaptation, evolving continuously in response to technological advancements and deepening our understanding of tumor biology [1]. Lattice radiation therapy (LRT), including lattice-spatially fractionated radiation therapy (L-SFRT) and lattice-stereotactic body radiation therapy (L-SBRT), is the latest advancement in managing bulky and complex tumor geometries surrounding dose-limiting critical organs at risk (OAR). Although its initial iterations were observed in two-dimensional (2D) grid radiation therapy during the early days of radiation oncology, it has undergone significant transformations, particularly in the modern era of radiation therapy [2].

The re-emergence and refinement of lattice radiotherapy owe much to the leaps made in radiation oncology techniques and the increasing sophistication of the treatment apparatus. With the use of advanced tools such as multileaf collimators (MLCs) and jaw-tracking methods, it is now possible to craft complex dose distributions that give rise to “peaks” and “valleys” of radiation intensity. These peaks, or “hot spots,” deliver high therapeutic doses directly to targeted tumor regions, known as lattice points. Conversely, the valleys provide rapid dose falloffs between these lattice points, ensuring minimal exposure to adjacent tumor volumes and normal tissues [3,4].

Understanding the efficacy of lattice radiotherapy is not just the technological capability to sculpt these dose landscapes but also a fundamental radiobiological rationale. The bystander effect, wherein non-irradiated cells manifest radiation-induced changes due to signals received from neighboring irradiated cells, and the abscopal effect, where distant non-targeted tissues exhibit a response to localized radiation, are central to this rationale [5,6]. Both effects challenge the traditional concepts of radiation dose delivery and distribution, opening avenues for therapeutic strategies that leverage these unique cellular responses.

This powerful combination of advanced technology and radiobiology has placed lattice radiotherapy at the forefront of efforts to achieve optimal tumor control with minimal harm to the surrounding tissues. As we continue to explore its potential, we aim to shed light on how L-SFRT and L-SBRT can revolutionize our approach to malignancies that have historically posed therapeutic challenges.

Here, we present two clinical scenarios that highlight the diversity of challenges and therapeutic options in oncology. The first patient, a 63-year-old male, had extensive non-small cell lung cancer (NSCLC) and was diagnosed with stage IIIB disease. Histopathological examination of the lung mass revealed a moderately to poorly differentiated squamous cell carcinoma. The second case involved a 61-year-old male diagnosed with high-grade papillary urothelial carcinoma in the left renal pelvis classified as stage IIIA. This was a recurrence after a nephroureterectomy, followed by three cycles of adjuvant cisplatin/gemcitabine chemotherapy. We have provided a detailed account of the clinical presentation, interventions, technical approaches, outcomes, and follow-up for each case.

## Case presentation

Case 1

Clinical Presentation

A 63-year-old male with a smoking history of 30 years initially presented with fatigue, weight loss, shortness of breath, and diarrhea. Routine blood tests indicated an elevated white blood cell count, prompting a referral to the emergency room. He also reported an unintentional weight loss of 20 pounds and a persistent cough. Computed tomography (CT) scan of the chest, abdomen, and pelvis displayed a 6 cm mass in the right upper lobe of the lung, suggestive of primary pulmonary malignancy, accompanied by right hilar lymphadenopathy. A biopsy of the lung mass revealed a moderately to poorly differentiated squamous cell carcinoma. Magnetic resonance imaging (MRI) of the brain showed no signs of intracranial metastases. A subsequent positron emission tomography (PET)-CT scan, on May 25, 2022, depicted a large, markedly avid mass in the right upper lung (RUL), suggesting primary lung cancer. Other findings included a few subcentimeter pulmonary nodules of ambiguous origin and low standardized uptake value (SUV) (2.2) mediastinal lymph nodes that were possibly metastatic. The patient then temporarily lost contact due to insurance issues, but when he returned for a checkup on September 7, 2022, a restaging PET-CT scan displayed the progression of the thoracic disease.

Case Management and Treatment Design

The patient was diagnosed with stage IIIB T4N2M0 non-small cell lung cancer of the right upper lung. Further testing confirmed the absence of actionable mutations. As a result, a comprehensive treatment plan incorporating chemoradiation with immunotherapy has been devised. Starting on September 9, 2022, he underwent weekly cycles of carbo/taxol for six weeks, combined with biweekly cycles of durvalumab for a total of 26 cycles. Radiotherapy was initiated on October 5, 2022. On the first day, a single fraction of 15 Gy was delivered to the lattice points within the large RUL mass, whereas the remaining thoracic tumor received 2 Gy. From the second day, standard 2 Gy fractions were administered for the next 29 fractions. There was a significant reduction in tumor volume by fraction 11, from a gross tumor volume (GTV) of 301.93 cm^3^ to 128.5 cm^3^, and re-planning was performed with the re-simulated CT images. The patient completed the treatment regimen on November 20, 2022, without experiencing any grade 2 toxicity and continued with durvalumab as scheduled, with the most recent session on August 8, 2023.

Treatment Planning and Delivery

The patient underwent simulation in a head-first supine position on a wingboard using the headrest. The vac-bag was used to mold the patient’s form with both arms raised. A contrast four-dimensional computed tomography (4D-CT) simulation captured the axial images with a slice thickness of 2.5 mm. GTV was delineated through PET-CT registration with the simulation CT, measuring an initial volume of 301.93 cm³. A planning target volume (PTV) margin of 7 mm was applied in all directions, resulting in a volume of 550.9 cm³. Five lattice spheres with diameters of 1 cm were created within the GTV. The separation between the surfaces of the adjacent spheres was maintained at 1.5 cm. These spheres were strategically positioned within hypoxic regions, ensuring that they were at least 1 cm from any organ at risk. The total volume of the lattice spheres was 6.7 cm³, which was 2.2% of the entire GTV.

**Figure 1:**
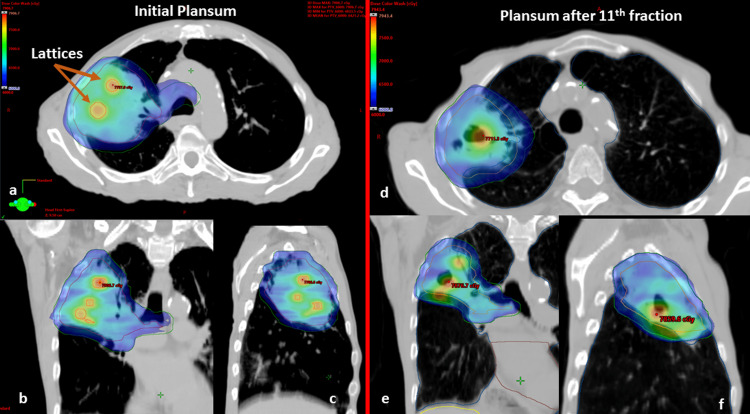
Left: Initial cumulative plan sum showing the 60 Gy color wash in blue to the entire PTV (lattice spheres were highlighted with arrows). Right: Cumulative plan sum showing 60 Gy isodose color wash (blue) after the re-simulation at fraction 11. a, b, and c: axial, coronal, and sagittal planes (initial plan sum), d, e, and f: axial, coronal, and sagittal planes (plan sum after 11th fraction) PTV: planning target volume

For the radiation treatment, 15 Gy in a single fraction was prescribed for each lattice sphere. This was optimized without controlling any hot spots inside the lattices using the biological target generalized equivalent uniform dose (gEUD) optimization method in the Varian Eclipse 16.1 (Paolo Alto, CA, USA) treatment planning system (TPS). Lattice spheres were optimized with a target value for a gEUD of 15 Gy. For this optimization, the “a” was set to -40. In contrast, the OARs were optimized using an upper gEUD with an “a” parameter value of +40, ensuring control of the maximum hot doses. The treatment employed 6 megavoltage (MV) X-ray energy on a Varian IX Linear Accelerator (LINAC) machine equipped with a 120 millennium MLC. A secondary plan was formulated using two partial arcs, uniformly delivering 2 Gy per fraction to the entire PTV over 29 fractions. The total prescribed PTV dose was 60 Gy, and the cumulative plan view is presented in Figure 1.

**Figure 2:**
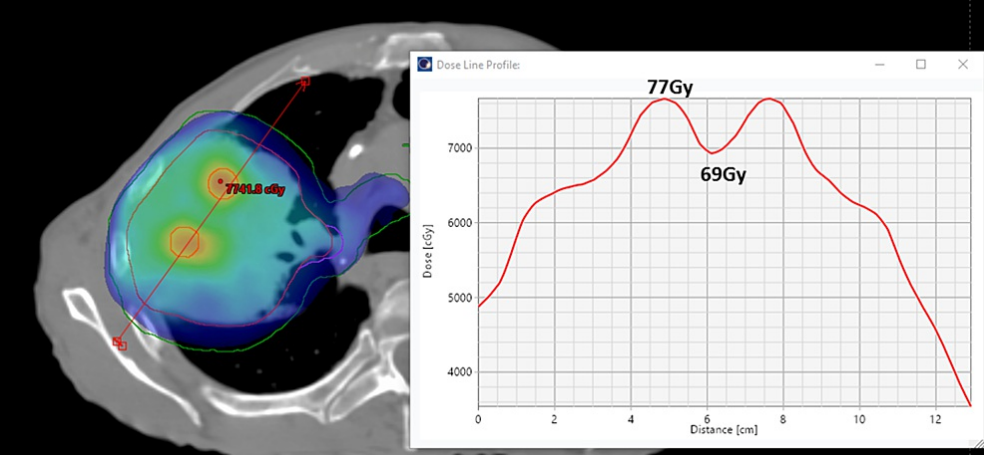
Initial cumulative plan sum showing the 60 Gy color wash (blue) to the entire PTV (dose line profiles depicting the peak (77 Gy) and valley (69 Gy) doses between the lattice spheres). PTV: planning target volume

The plan for the first fraction was to deliver 15 Gy to the lattice points, while the remainder of the PTV received an optimized 2 Gy, ensuring coverage of the entire PTV. The constraints set for the OAR were such that none should exceed a dose of 3 Gy. This was achieved with three partial arcs (ranging from 181° to 29°) using the volumetric modulated arc therapy (VMAT) technique. The OARs included the brachial plexus, esophagus, heart, spinal cord, and bronchial tree. The mean dose delivered to the lattices was 16 Gy. Notably, the highest recorded hot spot was 18.6 Gy within one of the lattice spheres, with a minimum valley dose of 9.5 Gy observed between two adjacent spheres, 7 mm apart (Figure 2). The first fraction ensured that 95% of the PTV was covered by the 2 Gy dose. The pixel maximum dose to the heart was recorded at 3.8 Gy, with all other OARs registered under 3 Gy. Both the cumulative 60 Gy/30 fractions and the individual lattice plan of the first fraction were meticulously evaluated for dose-volume histograms (DVHs), ensuring that no critical OARs reached unsafe maximum volume doses. The delineation of the OARs, targets, and plan evaluation was cross-verified by two radiation oncologists and an expert clinical physicist specializing in stereotactics. The total lung minus the GTV from the cumulative plan was V20 Gy = 15%, and all OAR doses were within the tolerance limit of the Quantitative Analysis of Normal Tissue Effects in the Clinic (QUANTEC) conventional dose constraints.

**Figure 3:**
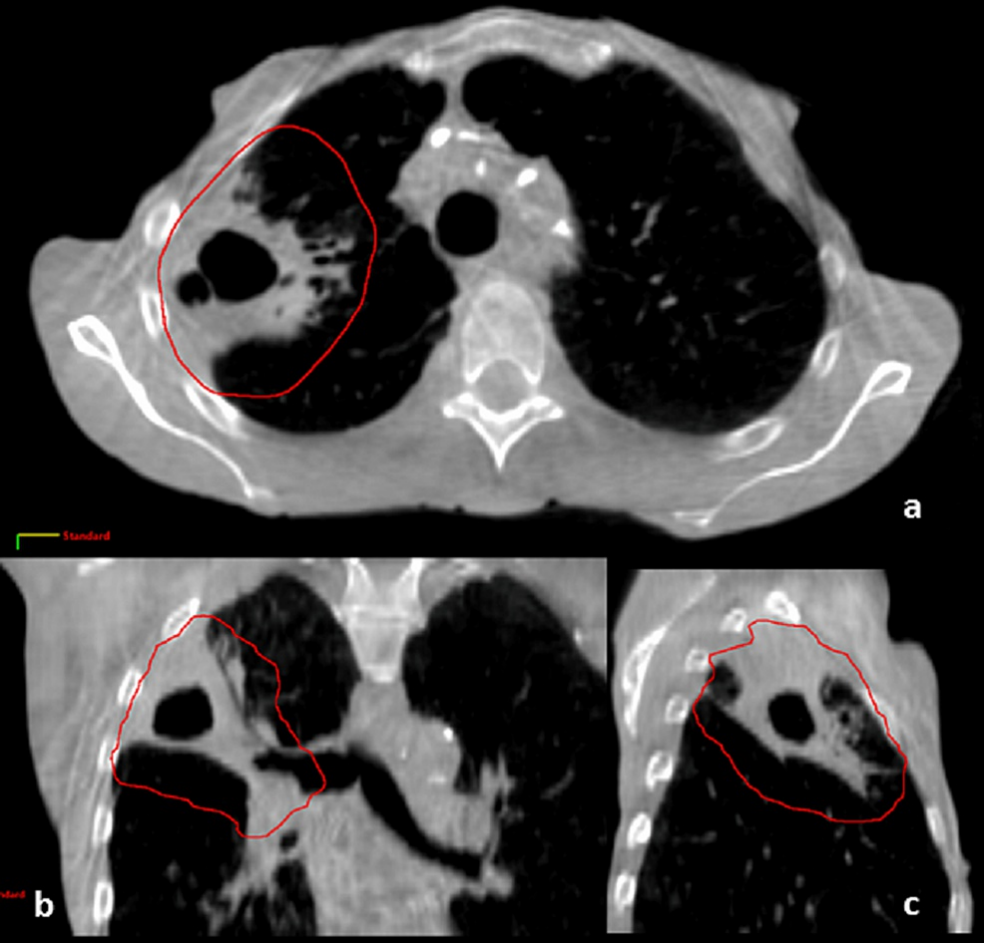
Final treatment CBCT images showcasing a considerable decrease in the size of the tumor. a, b, and c: axial, coronal, and sagittal planes (CBCT at fraction 30), outlined structure in red: original GTV at fraction 1 CBCT: cone beam computed tomography, GTV: gross tumor volume

The execution of the lattice plan involved a pretreatment cone beam CT (CBCT), which ensured accurate alignment of the lattice with the treatment geometry. Special attention was paid to the positioning of OARs near the lattice spheres, with the 8 Gy isodose line serving as a reference structure in CBCT. A noticeable reduction in GTV began on the second day of treatment and was prominently observed in the 11th fraction. This necessitated a re-simulation after the weekend break. The re-simulated GTV was recorded as 128.5 cm³, and the last CBCT (Figure 3) indicated a further reduction to 53 cm³ on the final treatment day.

Outcome and Follow-Up

**Figure 4:**
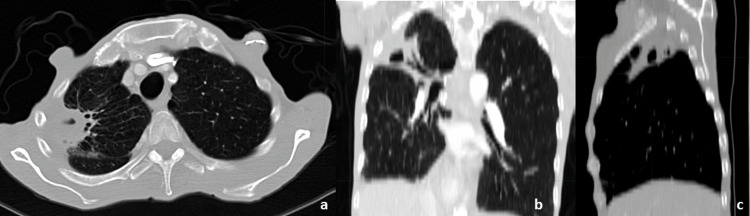
One-month post-radiation treatment follow-up CT scan. a, b, and c: axial, coronal, and sagittal image planes CT: computed tomography

**Figure 5:**
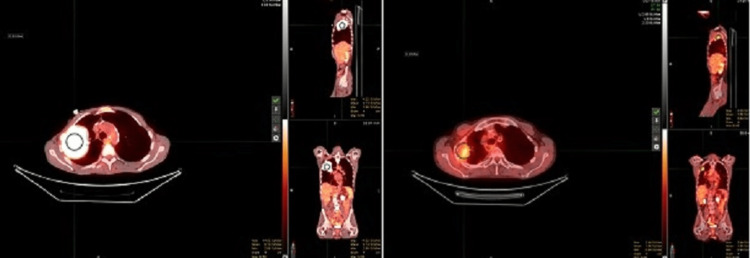
Left: Initial simulation PET-CT. Right: Recent follow-up PET-CT images showing complete response to L-SFRT. PET-CT: positron emission tomography-computed tomography, L-SFRT: lattice-spatially fractionated radiation therapy

The patient’s response to treatment was favorable, and no grade 2 toxicity was recorded (Figure 4). The patient’s overall health and performance status improved. Nine months after treatment, there were no symptoms of pneumonitis. A PET-CT scan on July 14, 2023, revealed a substantial reduction in the RUL mass, now a cavitating mass measuring 4 cm × 3.7 cm × 2.7 cm with a standardized uptake value (SUV) of 2.5 (Figure 5). There was also a decrease in the size of the mediastinal and hilar lymph nodes with no evidence of metastatic disease. Surveillance imaging will be used for continued follow-up every three months.

Case 2

Clinical Presentation and Management

In August 2021, a 61-year-old male presented to the emergency room with urinary difficulties. Imaging revealed a substantial left-sided renal mass. Consequently, he underwent a left nephroureterectomy on October 1, 2021. Pathological examination revealed invasive papillary urothelial carcinoma originating from the renal pelvis. Postoperatively, adjuvant chemotherapy with cisplatin and gemzar was initiated. By January 8, 2022, he had completed his third cycle with a carboplatin/gemzar combination due to renal insufficiency.

A follow-up CT of the chest, abdomen, and pelvis on May 20, 2022, showed post-nephrectomy changes for left renal pelvis transitional cell carcinoma. Alarmingly, there was an interval appearance of a 2.8 cm × 2.7 cm × 2.5 cm mass situated to the left of the aorta, right below the anticipated level of the left renal vasculature and just beneath post-surgical modifications. This finding is congruent with local recurrence. No distant metastases were identified within the thoracic, abdominal, or pelvic region. A left retroperitoneal/periaortic biopsy performed on June 14, 2022, confirmed this as a carcinoma, indicating that the malignancy was consistent with metastasis or recurrence of urothelial carcinoma.

After evaluating the prospects and potential challenges of definitive stereotactic body radiotherapy, we decided to proceed with a five-fraction SBRT regimen that included the use of lattice points. This approach was chosen because of the close proximity of the duodenum and small bowel to isolated recurrence. Radiation treatment was completed on July 14, 2022.

Treatment Planning and Delivery

Contrast 4D-CT simulation was conducted with the patient in the head-first supine position. A vac-bag was used to mold the patient’s form. The VMAT treatment plan with two full arcs for L-SBRT was crafted using Eclipse 16.1 TPS and was executed with the Varian IX Linear Accelerator, 6 MV X-ray energy, and 120 millennium MLC. The measured GTV was 25.5 cm^3^. The planning target volume (PTV) was derived by expanding the clinical target volume (CTV) by 5 mm, resulting in a volume of 60 cm^3^. The PTV was prescribed as 40 Gy in five fractions, and the GTV was 50 Gy in five fractions, with a lattice point dose prescription of 60 Gy in five fractions.

**Figure 6:**
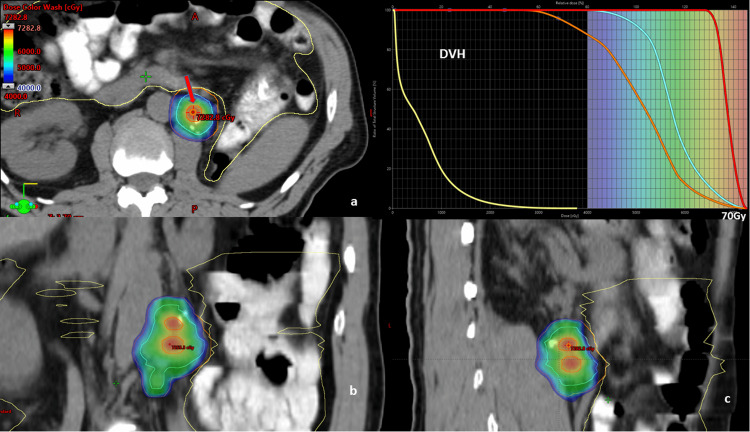
L-SBRT treatment plan depicting the 40 Gy isodose color wash to the PTV, and the lattice point is highlighted with an arrow. DVH: cyan: GTV, red: lattice spheres, orange: PTV, yellow: bowel structures a, b, and c: axial, coronal, and sagittal planes L-SBRT: lattice-stereotactic body radiation therapy, PTV: planning target volume, DVH: dose-volume histogram, GTV: gross tumor volume, PTV: planning target volume

Two lattice spheres, each 1 cm in diameter, were formed within the GTV. Care was taken to ensure that these spheres were at least 1 cm away from any bowel structure to minimize radiation exposure to this sensitive organ. The mean dose delivered to the lattice spheres was 68.8 Gy, whereas the maximum pixel dose reached was 72.8 Gy. During the optimization, this dose is not explicitly constrained. The gEUD optimization method was utilized. Lattice spheres were optimized with a target value for a gEUD of 60 Gy. For this optimization, the “a” was set to -40. In contrast, the OARs were optimized using an upper gEUD with an “a” parameter value of +40, ensuring control of the maximum hot dose to the bowel structures. GTV was adequately covered at a dose of 50 Gy. The remaining PTV also received 40 Gy, except for the regions overlapping with the bowel. In these areas, the dose was limited to 35 Gy to ensure that the bowel, the primary dose-limiting organ in this context, was protected from excessive radiation. The DVH is shown in Figure 6. Bowel structures met the institutionally developed SBRT dose-constraint volume of bowel structure of 0.035 cm^3^ receiving less than 35 Gy. The plan was delivered with pretreatment CBCT, and bowel structures and lattice point positions were checked for all five fractions.

Outcome and Follow-Up

**Figure 7:**
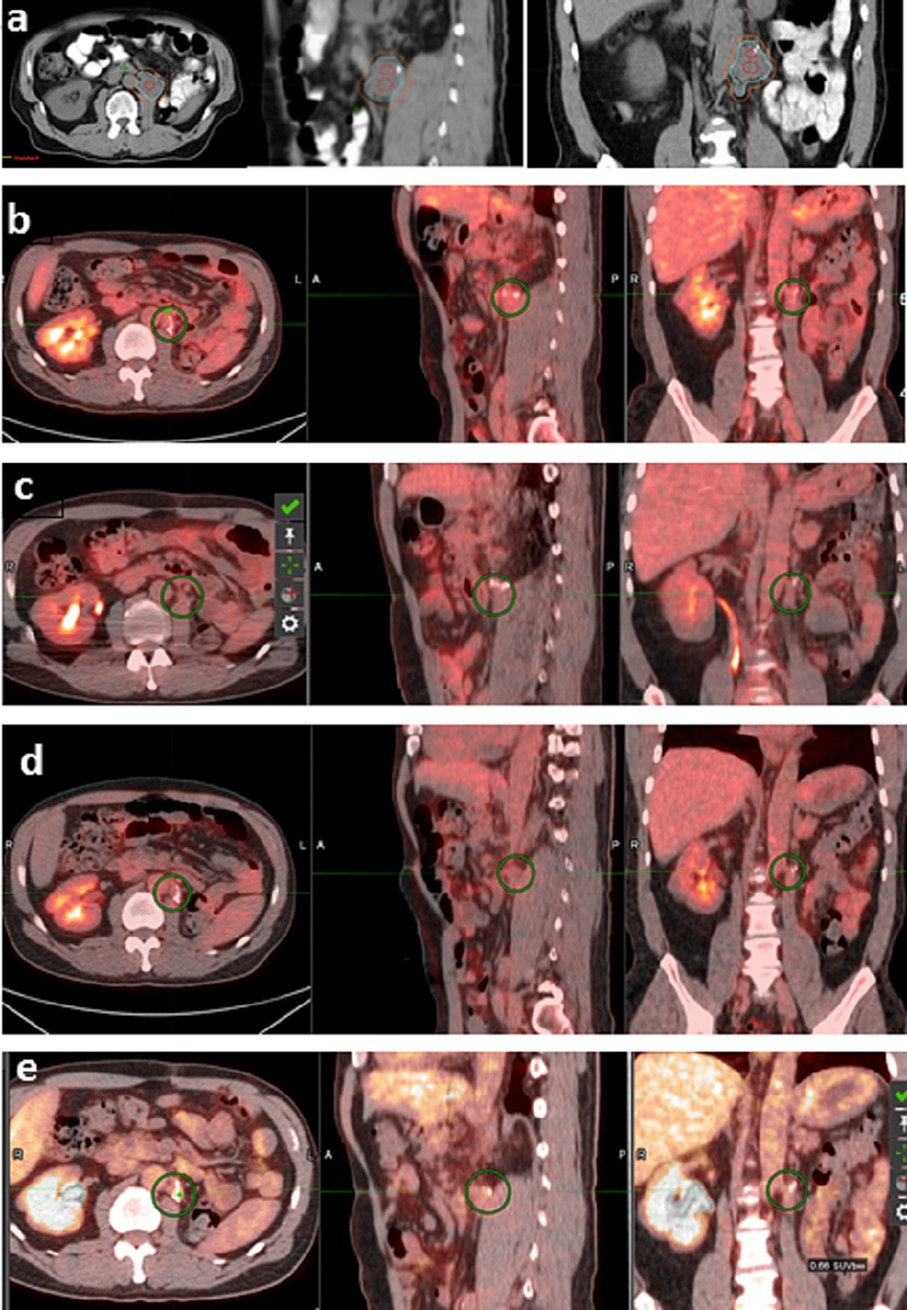
Sequential decrease in tumor size from PET-CT image fusions (axial, sagittal, and coronal) around the irradiated bed during the follow-ups. a: June 2022 treatment planning CT, b: August 2022 post-treatment, c: November 2022, d: February 2023, e: June 2023 PET-CT: positron emission tomography-computed tomography

Subsequent PET-CT imaging on August 30, 2022, post-radiation revealed a decreased size (1.5 cm × 1.5 cm) of the left periaortic soft tissue density adjacent to the left renal vasculature with only mild uptake of 2.6 SUV. In addition, no signs of disease progression were observed. Clinically, the patient demonstrated an excellent initial response, with no discernible treatment-related adverse events. During periodic three-month follow-ups, a consistent reduction in tumor size and mild uptake were noted (Figure 7). The patient remained in good health, without any signs of distant metastasis. The most recent follow-up on June 15, 2023, recorded a PET SUV of 2.3, and the patient’s condition remained stable and positive.

## Discussion

In this case report, the effectiveness and safety of 3D lattice radiation therapy were demonstrated through two clinical scenarios: a significant lung mass and a challenging dose-limiting situation involving an organ at risk. Both cases illustrate the potential of tailored radiotherapy approaches to escalate the prescription dose regardless of the complexity arising from larger tumor volumes and intricate organ-at-risk configurations. While the documentation is based on a one-year follow-up, the aim is to highlight the early experiences of customized lattice radiation therapy techniques and serve as a guide for the broader radiation oncology community.

A recent systematic review conducted by Iori et al. [7] evaluated the safety and efficacy of lattice radiation therapy for the treatment of bulky tumors. This study highlighted a limited amount of conclusive evidence and emphasized the need for more comprehensive research to fully understand the potential of LRT in managing large lesions.

A case report study presented by Amendola et al. [8] explored the efficacy of lattice radiotherapy in treating a 72-year-old patient with stage IIIA non-small cell lung cancer (NSCLC) combined with chemotherapy. Over a six-year follow-up period, the study demonstrated a complete response with minimal toxicity, indicating the capability of LRT in the management of advanced lung cancer.

Another study conducted by Amendola et al. [9] evaluated the safety and effectiveness of lattice radiotherapy for treating 10 bulky non-small cell lung cancer tumors. The study demonstrated that LRT was both safe and effective, with no significant toxicity observed, and highlighted the need for further clinical research.

Several small-scale studies [10-13] conducted across various tumor locations have demonstrated the safety and effectiveness of LRT for both palliative and definitive treatment of large tumors.

A recent clinical practice survey on lattice radiation therapy by Mayr et al. [14] showed that lattice-based treatment modalities are gaining popularity. However, the survey also highlighted the need for more standardized protocols and extensive clinical research to fully harness their potential.

In this light, our case report contributes a foundational step toward the said standardization, offering insights into plan evaluation and lattice placement strategies. As we continue to advance in the domain of LRT, future research should focus on its applicability in more complex cases and its long-term efficacy and safety. Additionally, there is a potential for LRT to be combined with immunotherapy, which deserves further exploration and could open a new chapter in personalized oncology treatments.

## Conclusions

Our early experience with lattice radiation therapy highlights its potential as a safe and effective therapeutic approach for treating large tumors, particularly when adjacent OARs present challenges. Considering its relative novelty and the progress of the latest systemic therapies, as well as the potential for combination with LRT, this report emphasizes the need for extensive research and multicentric trials to comprehensively evaluate its merits and limitations in diverse clinical settings.
